# Comparison of Uphold^TM^ Vaginal Mesh Procedure with Hysterectomy or Uterine Preservation for the Treatment of Pelvic Organ Prolapse

**DOI:** 10.1038/s41598-018-27765-8

**Published:** 2018-06-21

**Authors:** Chin-Ru Ker, Kun-Ling Lin, Zi-Xi Loo, Yung-Shun Juan, Cheng-Yu Long

**Affiliations:** 1 Department of Obstetrics and Gynecology, Kaohsiung Medical University Hospital, Kaohsiung City, Taiwan; 2 Department of Urology, Kaohsiung Medical University Hospital, Kaohsiung City, Taiwan

## Abstract

Hysterectomy is the empirical treatment for female pelvic organ prolapse (POP) without robust evidence to prove its efficacy. Uphold transvaginal mesh (TVM) system is an innovated device with smaller area, superior anterior/apical support with single incision to treat POP. The prospective cohort study aims to evaluate Uphold TVM’s performance with or without concomitant hysterectomy. Inclusion criteria specify patients suffering from stage 2 or more anterior/apical prolapse without previous hysterectomy. Exclusion criteria specify those with contraindications to uterine preservation, such as leiomyomas, adenomyosis, endometrial hyperplasia, abnormal uterine bleeding, postmenopausal bleeding, cervical dysplasia, receiving tamoxifen treatment, family history of gynecology cancer, or colon cancer and incapability to be routinely followed. Thirty patients are recruited in the hysterectomy group and 66 patients in the hysteropexy group. The results demonstrate that patients with Uphold mesh only experience longer vaginal length, shorter operation duration, less blood loss and less post-operation pain. The performance in anatomical correction, lower urinary tract symptoms improvement, de novo dyspareunia, urodynamic study parameters and mesh extrusion rates are comparable with or without uterus preservation. The information is useful in pre-operation counseling, when the patient can make an educated choice whether or not to receive concomitant hysterectomy.

## Introduction

Synthetic mesh once thrived in treating female pelvic organ prolapse (POP) for its effectiveness since its introduction in 1990s. As high as 41% of all prolapse procedures used synthetic mesh, reported by Medicare beneficiaries in the United States^1,2^. However, the device tumbled as Food and Drug Administration (FDA) of the United States issued a warning in 2011 questioning its long-term safety^3^. Media condemnation, legal issues and drop in marketing ensued. Regardless, Uphold Transvaginal Mesh (TVM) system (Boston Scientific, Natick, MA, USA) sustained to be the only mesh currently available in the United States. The innovative kit proclaimed superiority for smaller mesh area that reduces extrusion rates and improved support in the horizontal axis resulting in less prolapse recurrences. The insertion procedure was achieved in a single small vertical incision at anterior vaginal wall. Both short-term and medium-term reports revealed anatomical success rate more than 92% (100% for the apical compartment), reoperation rate less than 7.5% and mesh erosion rate of 2.5%^4,5^. Whether concomitant hysterectomy with this mesh resulted in differential proplase repair outcomes became a wonder.

Hysterectomy is the empirical treatment of choice for POP. Some advocate that concomitant hysterectomy reduces risks of cervical elongation and prolapse recurrence; it also prevents future cervical or uterine pathology^6,7^. However, Bonney from the 1930s argued that the descended uterus was a passive result of the causatively weakened pelvic floor. The extensiveness of neurovascular injuries from pelvic floor destruction of hysterectomy might surpass the benefits of repositioning the vagina. In fact, previous studies failed to demonstrate less prolapse recurrence rates in the concomitant hysterectomy subjects^8^. Furthermore, higher demands on life quality, self-esteem and sexuality by patients in modern societies have turned the tide, favoring uterine conservations^9^. With the aid of TVM that provides additional pelvic floor strength, the need of concomitant hysterectomy became even more dubious. Ultimately, the choice of hysterectomy or hysteropexy was determined through shared decision-making between the patient and her surgeon, after excluding those with previous mesh prolapse repair, cervical dysplasia, chronic pelvic pain, pathologic uteri or incapability to comply for regular surveillance.

Previous studies on concomitant hysterectomy during TVM insertion used earlier mesh products, namely Nasca^10^, Perigee, Apogee^11^, Elevated system^12^ and Prolift^7^. These reports varied in sample sizes (31–142 patients), mesh used and followed up periods (8.9–54 months), but they shared similar findings. Primary outcome with POP-Q found all parameters comparable between hysteropexy and hysterectomy groups, except for longer total vaginal length in the hysteropexy patients. Secondarily, hysteropexy groups experienced less operation time, less blood loss, shorter hospital stay and less mesh extrusion rates^7,10–12^. Similar findings were reported by Forde in his study with 1,601 women and follow-up period up to 3 years^13^. A systematic review and meta-analysis in 2017 with 11 publications reached agreeable conclusions^14^. The claimed cure rates ranged from 75% to 98%, and all prolapse recurrence cases were noted in the hysteropexy groups. It is of interest, and thus the objective of the current study, how Uphold mesh compares to these previous mesh studies, particularly in the outcome comparison between hysteropexy and hysterectomy.

## Materials and Methods

This is a prospective cohort study, initiated since October 2015 to September 2016, recruiting patients acquiring Uphold TVM (Boston scientific, Marlborough, MA) that reconstructs pelvic floor and repositions prolapsed pelvic organs. Patients with anterior and/or apical compartment prolapse scored by Pelvic Organ Prolapse Quantification System (POP-Q) stage 2 or more were included. We excluded patients who had previously received hysterectomy, lost follow up or had incomplete data. Pre-operation counseling pertaining to uterus sparing, procedures, safety, efficacy and potential complications were given. Contraindications to uterus conservation included confirmed or high likelihood of uterine or cervical pathology, such as leiomyomas, adenomyosis, endometrial hyperplasia, abnormal uterine bleeding, postmenopausal bleeding, cervical dysplasia, receiving tamoxifen treatment, family history of gynecology cancer, or colon cancer, and incapability to be routinely followed. Patients without the above-mentioned conditions chose between uterus preservation or concomitant hysterectomy in addition to TVM implantation. The option of concomitant midurethral sling insertion was offered to the patients who suffered from overt stress urinary incontinence or occult urodynamic stress incontinence, as detected in the urodynamic study. Ultimately, the patient made the educated decision whether or not a sling was added. One hundred and twenty-seven patients had entered this study. Thirty-one patients were excluded due to previous hysterectomy or incomplete medical records. A total number of 96 patients’ data were assembled and analyzed: 30 patients received concomitant hysterectomy when implanting Uphold TVM and 66 patients received Uphold mesh only (Hysteropexy group). Informed consent was obtained from all of our patients.

All procedures were performed by two experienced urogynecologists at a single center. Before implanting the mesh, a vertical incision at the anterior vaginal wall was made from the point below the bladder neck to the lowermost part of the prolapse. Diluted vasopressin (Pitressin, Parke Davis Division, Warner Lamber Canada Inc., Canada) solution was applied subcutaneous to reduce bleeding. With the Allis forceps securing incision margins, full-thickness blunt dissection was done for the pubo-cervical fascia laterally until reaching the sacrospinous ligaments. Dissection with 1–2 finger breadths further down from the ischial spines towards sacrum was done. The 2 legs of the Uphold mesh was attached to the bilateral sacrospinous ligaments with the aid of the Capio suture capturing device. The mesh was attached to the native vagina tissue with PDS 2-0 (Ethicon, NJ, US), followed by anterior colporrhaphy using Vicryl 3-0 (Ethicon, NJ, US). The legs of the mesh were pulled simultaneously at the same pace until the prolapse was reduced to its natural position. Excessive legs were removed. Complete vaginal wound closure with Vicryl 3-0. A roll of vaginal gauze for compression was applied and foley catheter inserted for 48 hours.

The primary outcome is prolapse reduction rate assessed objectively by POP-Q system. Recurrence is defined as POP-Q stage 2 or more found at any follow-up visit. Secondary outcomes included operation time (from first incision to last stitch), blood loss, intra-operative complications (bladder injury, rectal injury), post-operative complications (pain score, urinary tract infection, voiding dysfunction, perineal hematoma, de novo dyspareunia, mesh extrusion rate), urodynamic study performance, subjective satisfaction assessed with questionnaires, such as Overactive Bladder Symptom Scores (OABSS), Urogenital Distress Inventory (UDI-6), Incontinence Impact Questionnaire (IIQ-7), International Consultation on Incontinence modular Questionnaire (ICIQ), and Pelvic Organ Prolapse Distress Inventory 6 (POPDI-6). Data were collected before the procedure, followed by 1, 3, 6, 12 months and then semiannually after the operation. Mean follow-up duration was 6 months. Statistical analysis was performed using Student’s t test, Chi-squire test, paired t-test, Fisher’s exact test, Mann-Whitney U test, or Wilcoxon signed rank test for continuous variables, and the McNemar’s test for categorical variables. A difference was considered statistically significant when P < 0.05. The study protocols were approved by the Institutional Review Board of Kaohsiung Medical University Hospital, by which relevant guidelines and regulations were followed accordingly.

### Data availability

The datasets analysed during the current study are available from the corresponding author on reasonable request.

### Ethical Approval and Informed Consent

Ethics approval by the Institutional Review Board of Kaohsiung Medical University Hospital had been obtained for data analysis.

## Results

Characteristics of the tested groups were comparable, as revealed in Table 1. The mean age of our study groups was 66 years old, with a history of mean parity of 2.8–2.9 times and mean body mass index of 25.2–24.3. Five (16.7%) and eight (12.1%) patients received concomitant posterior colporrhaphy in the hysterectomy and hysteropexy groups respectively. Six (20%) and fifteen (22.7%) patients received concomitant mid-urethral sling procedures in the hysterectomy and hysteropexy groups respectively. The rates of concurrent posterior colporraphy and midurethral procedures were comparable in both groups.

**Table 1:** Clinical background of patients with pelvic organ prolapse in both groups. Data are given as mean ± standard deviation, mean [range], or n (%).

	Hysterectomy (n = 30)	Hysetropexy (n = 66)	P values
Mean age (years)	66.1 ± 9.0	66.6 ± 7.8	0.79*
Mean parity	2.9 ± 1.0	2.8 ± 1.1	0.84*
Mean BMI (kg/m^2^)	25.2 ± 3.6	24.3 ± 2.5	0.23*
Menopause	30 (100.0)	65 (98.5)	1.00**
Current hormone therapy	6 (20.0)	13 (19.7)	0.97**
Current smokers	0	1 (1.5)	1.00^
Diabetes mellitus	10 (33.3)	18 (27.3)	0.55**
Hypertension	15 (50.0)	37 (56.1)	0.58**
History of POP repair	0	3 (4.6)	0.55^
**Procedures in this study**			
Anterior colporraphy	30 (100)	66 (100)	1.00^
Posterior colporraphy	5 (16.7)	8 (12.1)	0.54**
Mid-urethral sling	6 (20.0)	15 (22.7)	0.76**

BMI: body mass index; POP: pelvic organ prolapse; *Student’s t-test, **Chi-square test; ^^^Fisher’s exact test.

Objective assessment of post-operation success rate with POP-Q system showed significant improvement at Aa, Ba and C points in both groups, without significant difference between groups (Table 2). Patients in the hysterectomy group also had significant improvement at Bp point but shorter total vaginal length. Total vaginal length is the only significantly different parameter between groups (p = 0.024) in POP-Q evaluations. Only 2 (3%) recurrent prolapse cases were documented in the hysteropexy group. Subjective assessment of patients’ urination performance showed significant improvements in frequency, SUI, urge incontinence, incomplete emptying, hesitancy and nocturia in both groups, without significant difference between groups (Table 3). Sixteen out of 41 preoperative overt SUI patients received slinging, and 87.5% of them remained continent at 6-month post-operative follow ups. Only 68% remained continent for those with preoperative overt SUI but did not receive slinging. On the other hand, 55 patients did not complain SUI preoperatively. Five of them received slinging for occult SUI detected by urodynamic studies and all of them remained continent at follow ups. For those who were continent preoperatively and did not receive slinging, 88% remained continent postoperatively (Table 4). Objective evaluation of patients’ urination performance with urodynamic study showed significant improvements in maximum flow velocity (Qmax) and residual volume (RU) in both groups, without significant difference between groups (Table 5).

**Table 2:** Pelvic organ prolapse quantification (POP-Q) values in both groups before and after surgery. Data are given as median (range) or n [%].

POP-Q parameters (cm)	Hysterectomy (n = 30)			Hysteropexy (n = 66)			P*^,a^
	Pre-OP	Post-OP	P values	Pre-OP	Post-OP	P values	
Aa	3(−3~3)	−2 (−3~−1)	<0.001	1 (−2~3)	−2 (−3~0)	<0.001	0.75
Ba	5 (2~9)	−2 (−3~−1)	<0.001	3 (−2~10)	−2 (−3~1)	<0.001	0.54
C	4 (−2~10)	−8 (−10~−7)	<0.001	2(−5~11)	−8 (−13~−5)	<0.001	0.21
Ap	−1 (−3~3)	−2 (−3~0)	0.004	−2 (03~3)	−2 (−2~0.5)	0.50	0.30
Bp	2.5 (−2~8)	−2 (−3~0)	<0.001	−1.5 (−3~10)	−2 (−2~−0.5)	0.001	0.11
Tvl	10 (7~13.5)	8.8 (8~10)	<0.001	9.5 (7~12)	−2 (−2~−0,5)	0.57	0.024
Recurrent patients	0			2 [3.0]		1.00**	

Pre-OP: preoperative; Post-OP: postoperative; Tvl: total vaginal length. *Wilcoxon signed rank; **Fisher’s exact test; ^a^comparison between groups.

**Table 3:** Urinary symptoms of patients with pelvic organ prolapse in both groups before and 6 months after surgery. Data are given as n (%).

Symptoms	Hysterectomy (n = 30)			Hysteropexy (n = 66)			P*^,a^
	Pre-OP	Post-OP	P values	Pre-OP	Post-OP	P values	
Urinary frequency	20 (66.7)	5 (16.7)	<0.01*	33 (50.0)	17 (25.8)	<0.01*	0.56
SUI	11 (36.7)	4 (13.3)	0.016**	30 (45.5)	12 (18.2)	<0.01*	0.72
UUI	14 (46.7)	5 (16.7)	<0.01*	30 (37.5)	9 (13.6)	<0.01*	0.89
Incomplete emptying	23 (76.7)	2 (6.7)	<0.01**	56 (84.9)	8 (12.1)	<0.01*	0.22
Urinary hesitancy	21 (70.0)	3 (10.0)	<0.01**	46 (69.7)	10 (15.2)	<0.01*	0.32
Nocturia	22 (73.3)	12 (40.0)	<0.01*	45 (68.2)	25 (37.9)	<0.01*	0.93

SUI: stress urinary incontinence; UUI: urge urinary incontinence; *McNemar’s test, **Fisher’s exact test; ^a^comparison between groups.

**Table 4:** Comparison of postoperative continence rate in women with and without preoperative stress urinary incontinence.

Preoperative SUI	Surgery	Continenct rate
Yes (n = 41)	Uphold + sling (n = 16) Uphold − sling (n = 25)	87.5% 68%
No (n = 55)	Uphold + sling (n = 5) Uphold − sling (n = 50)	100% 88%

SUI: stress urinary incontinence.

**Table 5:** Urodynamic changes in both groups before and 6 months after surgery. Data are given as n (%) or mean ± standard deviation.

Parameters	Hysterectomy (n = 30)			Hysteropexy (n = 66)			P*^,a^
	Pre-OP	Post-OP	P values	Pre-OP	Post-OP	P values	
DO	8 (26.7)	3 (10.0)	0.063*	16 (24.2)	8 (12.1)	0.15*	0.90
Qmax (ml/s)	11.3 ± 6.0	16.7 ± 7.7	<0.01**	13.1 ± 5.4	16.0 ± 6.8	0.03**	0.85
RU (ml)	68.6 ± 28.3	32.4 ± 18.1	0.09**	109.1 ± 42.0	36.6 ± 12.4	<0.01**	0.11
FS (ml)	127.5 ± 55.8	137.9 ± 29.5	0.43**	135.0 ± 49.0	146.2 ± 62.9	0.49**	0.32
MCC (ml)	336.4 ± 105.6	342.6 ± 108.7	0.35**	318.9 ± 120.7	329.8 ± 106.2	0.25**	0.25
Pdet (cmH_2_O)	27.6 ± 12.8	27.2 ± 13.6	0.13**	37.7 ± 20.1	34.7 ± 12.1	0.42**	0.06
FUL (mm)	22.5 ± 6.9	22.7 ± 3.1	0.94**	25.0 ± 7.1	26.5 ± 4.7	0.10**	0.10
MUCP (cmH_2_O)	60.8 ± 29.5	63.5 ± 26.8	0.23**	56.6 ± 31.8	58.2 ± 28.6	0.32**	0.09

DO: detrusor overactivity; Qmax: maximum flow rate; RU: residual urine; FS: first sensation to void; MCC: maximum cystometric capacity; Pdet: detrusor pressure at peak flow; FUL: functional urethral length; MUCP: maximum urethral closure pressure; UCA: urethral closure area. *McNemar’s test; **Paired t-test; ^a^comparison between groups.

Patients in the hysterectomy group endured significantly longer operation time (78.0 ± 16.0 minutes v.s. 46.0 ± 20.6 minutes, p < 0.001), more blood loss (36.7 ± 18.1 ml v.s. 18.5 ± 6.9 ml, p = 0.015) and higher post-operation day 1 pain score (2.1 ± 1.0 v.s. 1.7 ± 0.8, p = 0.025) compared to those in the hysteropexy group (Table 6). No bladder injury, rectal injury or blood transfusions were observed in any patient. Urinary tract infection rates, voiding dysfunction, perineal hematoma, de novo or worsened dyspareunia rates were comparable between the groups. Only one vaginal extrusion (1.5%) was noted in the hysteropexy group.

**Table 6:** Intraoperative, postoperative and mesh-related complications of patients with pelvic organ prolapse in both groups. Data are given as n (%).

	Hysterectomy (n = 30)	Hysteropexy (n = 66)	P values
**Intraoperative complications**			
Operative time (minutes)	78.0 ± 16.0	46.0 ± 20.6	<0.001
Blood loss (ml)	36.7 ± 18.1	18.5 ± 6.9	0.015
Blood transfusion	0	0	
Bladder injury	0	0	
Rectal injury	0	0	
**Postoperative complications**			
Post-op day 1 VAS score	2.1 ± 1.0	1.7 ± 0.8	0.025
Urinary tract infection	4 (13.3)	11 (16.7)	0.77*
Voiding dysfunction	2 (6.7)	8 (2.1)	0.72*
Perineal hematoma	2 (6.7)	0	0.10**
De novo or worsened dyspareunia	3 (10.0)	2 (3.0)	0.17**
**Mesh complications**			
Vaginal extrusion	0	1 (1.5)	1.00**
Bladder extrusion	0	0	

*Chi-square test; **Fisher’s exact test.

## Discussion

Although there is insufficient evidence to support concomitant hysterectomy at the time of prolapse repair, more than17.2% cases of hysterectomy are due to uterovaginal prolapse annually in the United States^15^. Even higher incidence of hysterectomy performed for POP is quoted from 21–38.6% among Medicare beneficiaries from 1999 to 2009^1^. Similarly, 86.5–90.6% prolapse patients received hysterectomy in Taiwan from 1997–2007^16^. Conventional reasoning advocates that hysterectomy reduces prolapse recurrence by lessening weight exerted on the weakened pelvic floor. An observation of more prolapse recurrences in the hysteropexy group compared to that in the hysterectomy group is recorded in related studies during TVM placement, although no between-group comparisons were made to reach robust implications^7,11^. Interestingly, similar trend is noted in this current study with 2 (3%) recurrent cases in the hysteropexy group and none in the hysterectomy group. Larger sample size and longer follow-ups are needed to examine if hysteropexy indeed plays a role in prolapse recurrence.

Another advantage proposed for hysterectomy in treating POP is the reduced risks of potential future cervical or uterine malignancy. The incidence of unanticipated cervical or uterine malignancies from specimens obtained from uterovaginal prolapse related hysterectomy is rather low: 2.6% as reported by Frick *et al*., among which none is pre-menopausal and 13.3% is post-menopausal with abnormal uterine bleeding^17^. The benefit of prophylactic hysterectomy in all prolapsed women is questioned. Current advent of screening tools such as sonography, Papnicolaou smear and HPV tests should be helpful in identifying patients legitimate for prophylactic hysterectomy.

A trend towards uterine preservation in uterovaginal prolapse surgeries is apparent worldwide^1,6,12,16,18^, due to fertility desire, raising awareness of sexuality, body image, self-esteem, quality of life and cultural beliefs. The shift of women’s attitude is well demonstrated in Frick’s study, in which questionnaire surveys reveals 60% of women prefer to spare their uterus if equal surgical efficacy can be reached compared to concomitant hysterectomy^19^. Korbly goes further to show that 36% of women preferred uterus preservation even if it has less surgical efficacy^20^. Uterus preservation at the time of TVM placement is plausible as demonstrated by various studies with equal or non-inferior surgical outcomes compared to those in concomitant hysterectomy^10–12^. Cure rates range from 75% to 98%, with the advantages of longer vaginal length, shorter operation time, less blood loss and less post-operation pain, which are all in agreement with this current study. When Uphold TVM is placed for stage 2 or more uterovaginal prolapse patients, objective assessment in POP staging (with the exception of total vaginal length), subjective lower urinary tract symptoms and peri-operation complications were all significantly improved without a difference between groups. These results are analogous to studies of precedent meshes^7,21,22^.

Interestingly, reducing uterovaginal prolapse could improve urinary functions, particularly in detrusor overactivity and residual urine volume^21,23,24^. It is reasonable because by reducing urethral kicking, the patients urinate better with less residual urine and consequently, less distended bladder that induces detrusor muscle irritability. The assertion is supported by current study with Uphold mesh, an improvement in urinary peak flowrate reaches statistical significance for both hysterectomy and hysteropexy groups, without significant difference between groups. A post-TVM de novo SUI rate of 12% is detected, which is compatible to the 2–22% rate reported by previous studies^25^. The wide range might attribute to sample size, characteristics of patients included and inconsistent definition of de novo SUI from different studies. For the current study, patients with vault prolapse are excluded and those with occult SUI included. The reduction of prolapsed organs and therefore the relief of urethral obstruction are likely the reasons for de novo SUI, or in some cases, undetected occult SUI. The rationale is supported by the work of Kuribayashi *et al*., in which a correlation between the severity of POP and post-TVM de novo SUI is suggested^25^. The issue of post-TVM SUI is an area of interest and subject to further investigations by further studies.

One of the advantages of hysteropexy is preserved vaginal length, which could positively affect sexuality. De Vita *et al*. reported increased sexual activity by only 12.5% of the hysterectomy group, comparing to 95% identical or improved sexual quality and 1.3% de novo dyspareunia for the hysteropexy group using Gynemesh^22^. Similar satisfactory responses in sexual functions after hysteropexy were also reported by Jeng *et al*. and Dietz *et al*., although no comparison to hysterectomy cases was conducted^26,27^. On the other hand, Huang *et al*. reported comparable sexual performance between hysteropexy and hysterectomy groups when using Prolift system^7^. In this current study, 3 (10%) versus 2 (3%) de novo dyspareunia is observed in the hysterectomy and hysteropexy groups, respectively, without significant difference in between groups. This information might be helpful when offering pre-operation counselling.

Mesh extrusion is a major concern for concomitant hysterectomy at the time of TVM placement, with the reported increased odds of 13% vs 4% for the Perigee, Apogee study^11^, 13.8% vs 2% for the Elevate systems^12^, and 5-fold risk by Collinet *et al*.^28^. The reported exposure rate for Uphold mesh is 6.5%, with most patients not requiring surgical interventions^4^. In this current study, only 1 mesh extrusion (1.5%) from the hysteropexy group is reported; none is noted from the hysterectomy group. The sample size is too small to make between-group comparisons but should be re-examined as data accumulates. The performance is comparable to the reported 2–3.4% mesh erosion rate in laparoscopic sarcocolpopexy procedures^29,30^. Longer follow-up periods and larger sample size might be required to assess the true mesh extrusion rate, and thereby testifying if the small single-site incision design is indeed better as claimed.

We acknowledge our weakness of having small sample size, but this is the first series comparing the treatment efficacy between hysterectomy and hysteropexy using Uphold TVM system. Studies with larger sample size and longer follow up periods are needed for validate the results of this study.

In summary, Uphold TVM system has superior anatomical correction and less extrusion rates compared to precedent meshes. Similar to previous studies, patients experience longer vaginal length, shorter operation duration, less blood loss and less post-operation pain with hysteropexy. The performance in anatomical correction, lower urinary tract symptoms improvement, de novo dyspareunia, urodynamic study parameters and mesh extrusion rates are comparable with or without uterus preservation. The information is useful in pre-operation counseling, when pathologic or potentially pathologic cervix or uterus is excluded, the patient can make an educated choice whether or not to receive concomitant hysterectomy.
